# TFTenricher: a python toolbox for annotation enrichment analysis of transcription factor target genes

**DOI:** 10.1186/s12859-021-04357-4

**Published:** 2021-09-16

**Authors:** Rasmus Magnusson, Zelmina Lubovac-Pilav

**Affiliations:** School of Bioscience, Systems Biology Research Center, University of Skövde, Skövde, Sweden

## Abstract

**Background:**

Transcription factors (TFs) are the upstream regulators that orchestrate gene expression, and therefore a centrepiece in bioinformatics studies. While a core strategy to understand the biological context of genes and proteins includes annotation enrichment analysis, such as Gene Ontology term enrichment, these methods are not well suited for analysing groups of TFs. This is particularly true since such methods do not aim to include downstream processes, and given a set of TFs, the expected top ontologies would revolve around transcription processes.

**Results:**

We present the TFTenricher, a Python toolbox that focuses specifically at identifying gene ontology terms, cellular pathways, and diseases that are over-represented among genes downstream of user-defined sets of human TFs. We evaluated the inference of downstream gene targets with respect to false positive annotations, and found an inference based on co-expression to best predict downstream processes. Based on these downstream genes, the TFTenricher uses some of the most common databases for gene functionalities, including GO, KEGG and Reactome, to calculate functional enrichments. By applying the TFTenricher to differential expression of TFs in 21 diseases, we found significant terms associated with disease mechanism, while the gene set enrichment analysis on the same dataset predominantly identified processes related to transcription.

**Conclusions and availability:**

The TFTenricher package enables users to search for biological context in any set of TFs and their downstream genes. The TFTenricher is available as a Python 3 toolbox at https://github.com/rasma774/Tftenricher, under a GNU GPL license and with minimal dependencies.

**Supplementary Information:**

The online version contains supplementary material available at 10.1186/s12859-021-04357-4.

## Background

Transcription factors (TFs) hold a central role in the regulation of gene expression. There are numerous studies that identify human TFs that potentially regulate the gene expression of interesting processes, disease related mechanisms, etc. [1]. Important analyses of ATAC-Seq data, gene regulatory networks (GRNs), and expression quantitative trait locis (eQTLs) all revolve around TFs. Having identified a set of potentially important TFs, a logical next step in a bioinformatics analysis pipeline is to connect those TFs to downstream genes and, subsequently, to biological functions. There are several methods available to give biological context to sets of genes using underlying functional annotation databases, such as the Kyoto Encyclopedia of Genes and Genomes (KEGG) [2], Reactome [3], and Gene Ontology (GO) [4]. Annotations in such databases are typically matched to a set of genes, and the top overlapping annotations help to shed light on what functionalities the genes represent [5].

However, an analysis pipeline that goes from TFs to biological functions is facing at least two major pitfalls. First, TFs are themselves genes with a specific function—transcriptional regulation. Thus, by using the simple gene set enrichment analysis approach, we will usually not discover the functional patterns of downstream genes that these TFs regulate. If a set that exclusively contains TFs is used to extract, as an example, enrichments of GO-terms, the predominant associations will by definition be related to gene transcription. While this is an accurate annotation, we note that such an analysis gives little or no information about what downstream processes the TFs control. The second pitfall concerns statistical power. There are roughly 1,600 known human TFs, constituting only 5–8% of the human genome, which limits statistical power of such comparisons. To solve these hurdles, and to simplify annotation enrichment analysis of genes downstream of TFs, we present the TF target enricher (TFTenricher), a Python toolbox that maps TFs to their target genes, and calculates their overlap with genes sets in some of the most widely used annotation sets.

## Implementation

**Fig. 1:**
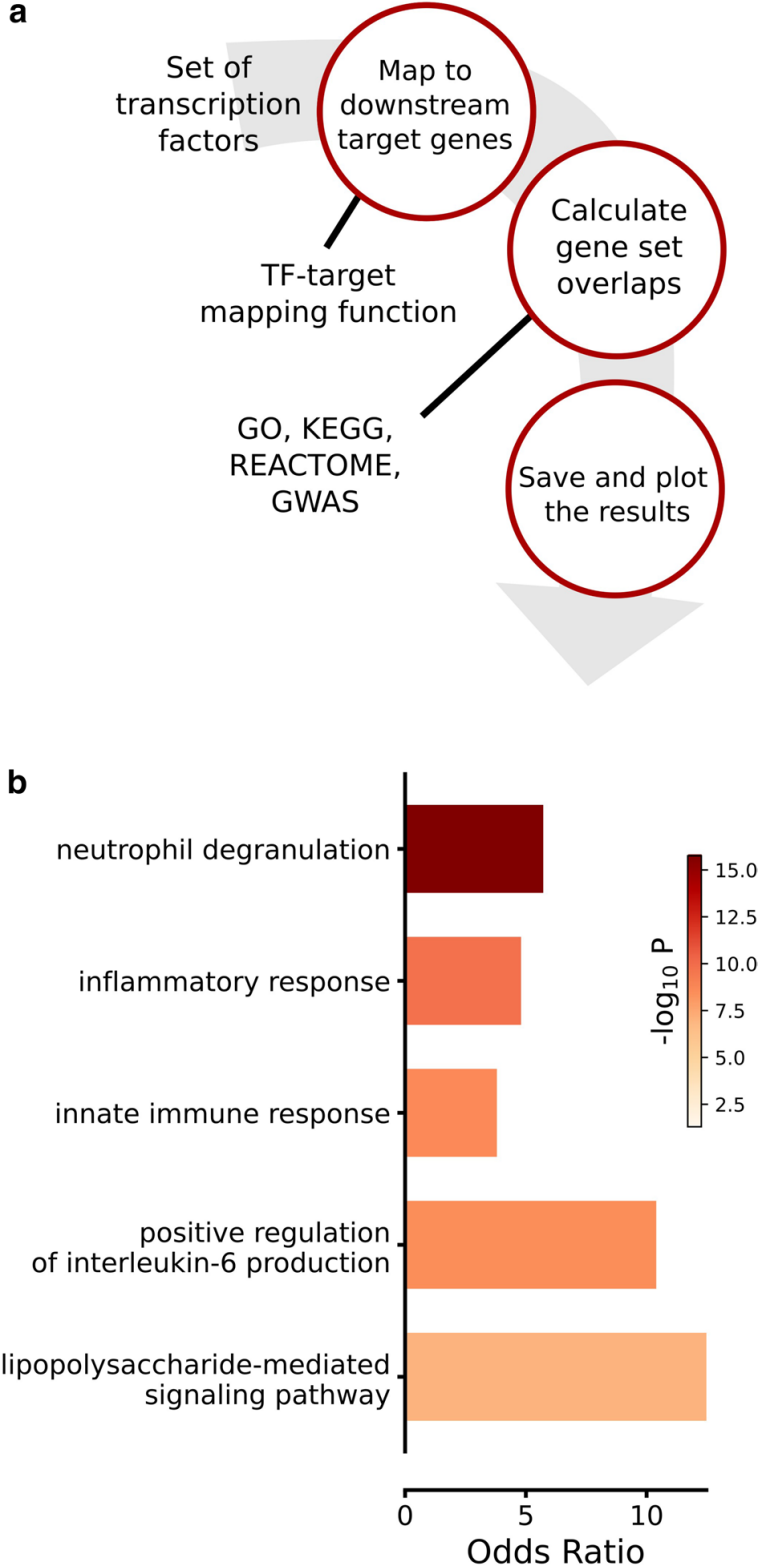
TFTenricher functionality. **a** Flowchart of the TFTenricher approach. A set of TFs are taken as input, which then by default are mapped to target genes using a pre-compiled TF-target coexpression matrix. The user can also use custom-built TF-target mapping functions. Next, the target genes are annotated with GO (default), KEGG, REACTOME, or diseases using the GWAS catalog. The user can also provide a custom gene set for overlap calculations. **b** A typical output of the TFTenricher in terms of GO enrichments, here when applied to multiple sclerosis-associated TFs (as found in Additional file 2)

The TFTenricher was developed in Python 3, under a GNU General Public License V3, and is, together with a user tutorial, available at https://github.com/rasma774/Tftenricher. The TFTenricher is dependent on just four of the most common Python packages, namely NumPy, Scipy, Pandas, and Matplotlib, which are included in most Python installations, allowing for an easy install. The default TFTenricher algorithm works in three distinct steps (Fig. 1a). First, it maps a user-defined list of TFs to putative downstream genes using lookup-tables of co-expression that comes included with the software. To date, the known human gene regulatory network remains incomplete, and the TFTenricher can use putative TF-target interactions that are either supplied by the user, or alternatively, built in to the TFTenricher. The built-in inference methods are, as of now, based on either the TRRUST [6] database, the STRINGdb [7], or a Pearson correlation coefficient matrix based on expression that was extracted from ARCHS4 database [8]. In the case of the TRRUST and STRINGdb, the input TFs are pooled together and the top *n* genes with the strongest associations are extracted. In the correlation-based target gene extraction, the genes’ absolute values of the correlation coefficients are summed, and the top *n* genes are returned. If the user chooses not to specify the input parameter *n*, a Monte Carlo-based function randomly draws TFs and the top ranking target genes are compared to random chance. Furthermore, the correlation matrix, which is the default inference method of TFTenricher, is based on data from > 100 k gene expression profiles, which makes it one of the most extensive co-expression analyses currently available.

The second step in the TFTenricher algorithm takes the mapped target genes and uses Fisher’s exact test to calculate the enrichments of gene sets annotated in, as per the choice of the user, KEGG, GO, REACTOME, the GWAS catalog, or alternatively, sets that are supplied by the user. The default is a GO enrichment of biological process, cellular component, and molecular function based on PANTHER GOslim [4]. Moreover, multiple testing correction is available using either a Bonferroni or Benjamini–Hochberg correction, or additional correction approaches as provided by the user. As a third and final step of the TFTenricher algorithm, the odds ratio and *p* values of the most enriched terms can be plotted (Fig. 1b), or saved to file.

## Results

### The TFTenricher increases power in TF-oriented annotation analyses

We analysed performance by randomly drawing transcription factors (TFs) from the Human Transcription Factors database [9], which annotates TFs based on a broad selection of popular databases. Moreover, we drew TFs ten times for each step of 50 in the range of 50–450 TFs. We set 450 TFs as an upper limit of this analysis, noting that 450 TFs exceed a quarter of all human TFs found in the database, and applied TFTenricher to the permutations. The TFTenricher completed calculations under 30 s in all permutations (Additional file 1).

We next analysed the performance of the TFTenricher when applied to differentially expressed TFs from a compendium of 21 diseases (Additional file 2). We found the TFTenricher to identify a median of 54 terms at a false discovery rate of 0.05, whereas applying TFTenricher on TFs only resulted in a median of 12 identified terms per dataset (Wilcoxon signed-rank test *p* < 0.006). We thus conclude that the TFTenricher markedly increases the statistical power of analyses of biological function. We also note that in our analysis, the top GO-terms of the TFs themselves invariably involved the regulation of transcription by RNA polymerase II, whereas the TFTenricher inferred disperse and biologically relevant annotations (Fig. 1b, Additional file 2).

### Correlation-based inference of downstream processes minimises false positive identifications

To date, there is no complete interaction map between human TFs and their target genes, and there are multiple available approaches to infer such interactions [10]. Whereas most such approaches infer bindings from specific datasets, we sought to include dataset-independent TF-target interaction maps. To this end we incorporated the TRRUST [6] and STRINGdb [7] databases and the gene expression correlation matrix developed by Lachmann et al. [8]. By applying the TFTenricher to 100 sets of random TFs we found the co-expression based TF-target inference method to result in considerably fewer false positive identifications, with on average 2.16 GO terms (Additional file 3). Furthermore, the majority of these GO terms were related to transcription, with the terms *mRNA splicing, *via* spliceosome*, and *mRNA processing* accounting for 57% of all identified terms. We speculate these identifications being due to the TFTenricher, by the nature of the correlation-based target gene inference, identifying genes that are involved in transcription without being TFs themselves. From these results we chose to make the co-expression based method the default setting of TFTenricher. However, we note that co-expression as a tool of gene regulatory inference is prone to several pitfalls [11], and as alternative data, e.g. massive and unbiased ChIP-Seq databases, become available, TFTenricher can easily be expanded to also include such data. Arguably all TF-target inference methods contain various drawbacks and we therefore built the TFTenricher to allow for independent TFtarget mappings supplied by the user.

## Conclusions

The bioinformatics community provides excellent tools to associate biological functions to sets of genes. However, when those genes are TFs, results will likely fail to detect the processes of genes that are regulated by the TFs. We present the TFTenricher, a Python tool that enables researchers to analyse biological function of genes that are downstream of a set of a priori interesting TFs. The TFTenricher enables users to perform enrichment analyses of gene set associations in several popular databases, all with a minimal set of dependencies.

## Supplementary Information

**Additional file 1**. TFTenricher run time. An analysis of the wall-clock time needed to run TFTenricher with default settings.

**Additional file 2**. The TFTenricher applied to differentially expressed TFs. An analysis of TFTenricher applied to differentially expressed transcription factors of 21 diseases.

**Additional file 3**. Sensitivity and specificity of transcription factor-target mappings. We tested the ability of TFTenricher to (1) identify specific GO-terms when only given the transcription factors of the associated genes in each set, and (2) to not give false identifications when applied to random sets of transcription factors.

## Data Availability

Project name: TFTenricher. Project home page: https://github.com/rasma774/Tftenricher. Operating system(s): UNIX-like systems. Programming language: Python 3.8. Other requirements: NumPy > 1.18.5, Pandas > 1.0.5, Matplotlib > 3.2.2, Scipy > 1.5.0. License: GNU General Public License V3. Any restrictions to use by non-academics: license needed. TFTenricher and implementations made freely available at https://github.com/rasma774/Tftenricher. The code used for the results in S2 and S3 are available at https://github.com/rasma774/TFTenricher_files.

## Abbreviations

eQTLs: Expression quantitative trait locis
GO: Gene Ontology
GRN: Gene regulatory network
KEGG: Kyoto Encyclopedia of Genes and Genomes
TF: Transcription factor
