# Supplementary material for: TFTenricher: a python toolbox for annotation enrichment analysis of transcription factor target genes
Source: BMC Bioinformatics. 2021 Sep 16;22:440. doi: 10.1186/s12859-021-04357-4 (PMC8444601; doi:10.1186/s12859-021-04357-4)
Supplement: Supplementary file 1 — Additional file 1. TFTenricher run time. An analysis of the wall-clock time needed to run TFTenricher with default settings. [file 12859_2021_4357_MOESM1_ESM.pdf]

Supplementary material S1

TFTenricher run time

We analysed the TFTenricher run time as a function of ingoing number of transcription factors (TFs). To this end, we used the default TFTenricher parameters, i.e. using gene correlations to annotate downstream genes and comparing these genes to GO annotations. Furthermore, we note that the correlation approach is the slowest mapping function implemented in the package.

We randomly drew transcription factors from the Human Transcription Factors database, Lambert et al. 2020, which annotates TFs based on a broad selection of popular databases. Moreover, we drew TFs ten times for each step of 50 in the range of 50-450 TFs. We set 450 TFs as an upper limit of this analysis, noting that 450 TFs exceed a quarter of all human TFs found in the database, and applied TFTenricher to the permutations. The TFTenricher completed calculations under 30 seconds in all permutations, with the median time to completion being <16 seconds. We observed a linear increase in run time as the number of TFs increased (Fig. S1).

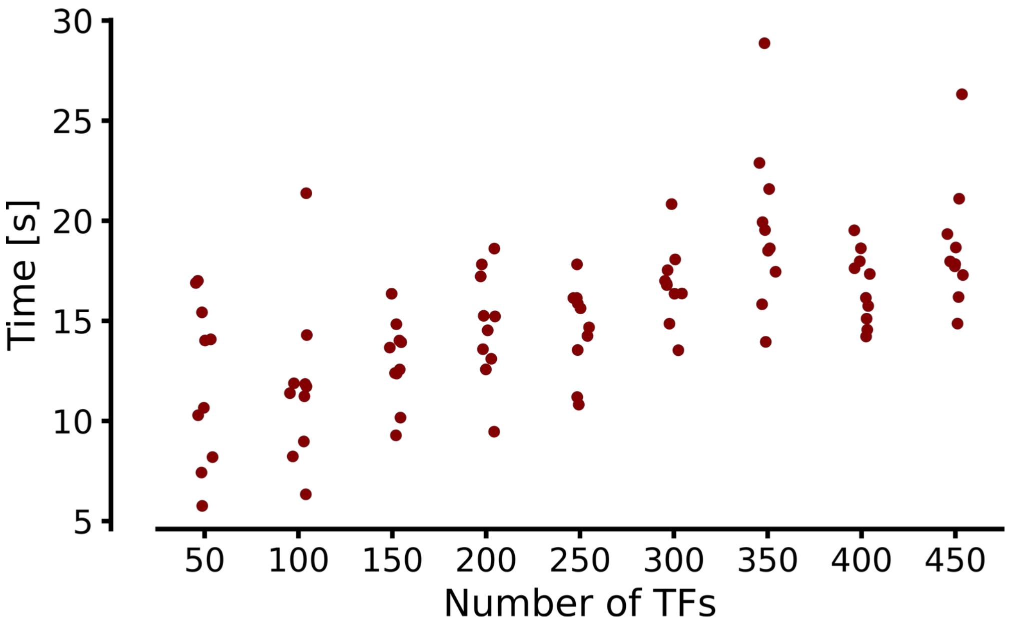

Fig. S1. Time to completion as function of ingoing TFs. We tested ten random permutations of TFs at each interval of 50 between 50 and 450, and found a linear increase in completion time of approximately 7 seconds when increasing the number of random TFs to 450 from 50.

References

Lambert SA, Jolma A, Campitelli LF, Das PK, Yin Y, Albu M, Chen X, Taipale J, Hughes TR, Weirauch MT. The Human Transcription Factors. Cell. 2018 Feb 8;172(4):650-665. doi: 10.1016/j.cell.2018.01.029. Erratum in: Cell. 2018 Oct 4;175(2):598-599. PMID: 29425488.
