# Supplementary material for: TFTenricher: a python toolbox for annotation enrichment analysis of transcription factor target genes
Source: BMC Bioinformatics. 2021 Sep 16;22:440. doi: 10.1186/s12859-021-04357-4 (PMC8444601; doi:10.1186/s12859-021-04357-4)
Supplement: Supplementary file 2 — Additional file 2. The TFTenricher applied to differentially expressed TFs. An analysis of TFTenricher applied to differentially expressed transcription factors of 21 diseases. [file 12859_2021_4357_MOESM2_ESM.pdf]

Supplementary material S2

The TFTenricher applied to differentially expressed TFs

To demonstrate the functionality of TFTenricher, we applied it to all available differential RNA-Seq studies of diseases from the Expression Atlas (n=21). By applying TFTenricher to the respective sets of differentially expressed TFs of each disease, we mapped TFs to downstream genes and analysed the respective GO term enrichments. Compared to GO enrichments on the TFs themselves, we found TFTenricher to identify a wider selection of enriched terms at a false discovery rate (FDR) of 0.05. Indeed, the median number of significant GO-terms at this FDR-level was 54, whereas the same analysis on the TFs showed a median of 12 identified GO terms per dataset (paired Wilcoxon signed-rank test $p<0.006$). We continued by analysing the corresponding results with the KEGG, REACTOME and GWAS annotation functions. In detail, the KEGG and GWAS-based analysis found a median of 0 annotations in both cases. The REACTOME-based analysis showed an increased number of identification from 2 to 6 when moving from TF to target-based inference ($p<0.008$). We thus hypothesise that TFTenricher increases the statistical power of analyses of biological function.

We designed TFTenricher with the hypothesis that annotating biological function to sets of TFs directly will bias the analysis towards identifications that revolve around the process of transcription itself – as opposed to the, arguably, more relevant downstream processes. We note that in our analysis, the top GO enrichments of the TFs themselves invariably involved the regulation of transcription by RNA polymerase II, whereas TFTenricher showed dispersed and biologically relevant annotations.
