# Supplementary material for: TFTenricher: a python toolbox for annotation enrichment analysis of transcription factor target genes
Source: BMC Bioinformatics. 2021 Sep 16;22:440. doi: 10.1186/s12859-021-04357-4 (PMC8444601; doi:10.1186/s12859-021-04357-4)
Supplement: Supplementary file 3 — Additional file 3. Sensitivity and specificity of transcription factor-target mappings. We tested the ability of TFTenricher to (1) identify specific GO-terms when only given the transcription factors of the associated genes in each set, and (2) to not give false identifications when applied to random sets of transcription factors. [file 12859_2021_4357_MOESM3_ESM.pdf]

Supplementary material S3

Sensitivity of the co-expression based TF-target mapping

There is to date no perfect mapping between transcription factors (TFs) and their target genes. TFTenricher maps TFs to target genes by analysing gene expression correlations across tens of thousands of RNA-Seq samples, as made available by Lachmann et al., 2018. While there are several other approaches to infer TF-to-target interactions (Marbach et al. 2012), whereof correlation-based methods are one of the most common, we built TFTenricher to take alternative and user-defined TF-target mappings as input. To test the default, correlation-based TF-target mapping approach in TFTenricher, we defined a test where we took all GO terms with >20 TFs and >20 target genes, used the TFs as input to TFTenricher and analysed the overlap between the putative target genes and the respective GO terms. We note that TFTenricher by default does not include the input TFs in the set of putative target genes. Furthermore, we also note that there is no guarantee that the TFs in one GO annotations are regulating the rest of the genes in the GO set, and we do therefore not expect a perfect overlap. Yet, we found 58 GO terms to meet the inclusion criteria, and of those 15 displayed a significant overlap with their respective putative target genes when the TFs were applied to TFTenricher (binomial test $p<1.2e-7$).

Specificity of the TF-target mapping

Mappings between transcription factors (TFs) and target genes will be subject to biases. We chose to study three different approaches to map TFs to target genes. In detail, we analysed the biases each approach was subject to by drawing 100 independent sets of 100 TFs each, and applied TFTenricher with mappings of the default gene-gene correlations (Lachmann et al. 2018), but also analysed the corresponding, built-in mappings using the TF-targets mappings in the TRRUST database (Han et al. 2018), and the protein-protein mappings of STRINGdb (Szklarczyk et al 2019). Moreover, we chose to study the top 200 downstream genes from each set, and calculated the overlap with GO, KEGG, REACTOME and GWAS enrichments (Fig. S3). In these 100 test sets, we observed the TRRUST and STRINGdb to give multiple false positive identifications after a Benjamini-Hochberg correction at FDR=0.05 in all comparisons. (TRRUST-GO mean: 599, TRRUST-KEGG mean: 22, TRRUST-REACTOME mean: 115, TRRUST-GWAS mean: 20, STRINGdb-GO mean: 491, STRINGdb-KEGG mean: 33, STRINGdb-REACTOME mean: 209, STRINGdb-GWAS mean: 7). The target genes inferred based on expression correlation, however, displayed notably fewer interactions passing a FDR correction (2.2 GO identifications, 0 KEGG, 0.3 REACTOME and 0 GWAS identifications on average).

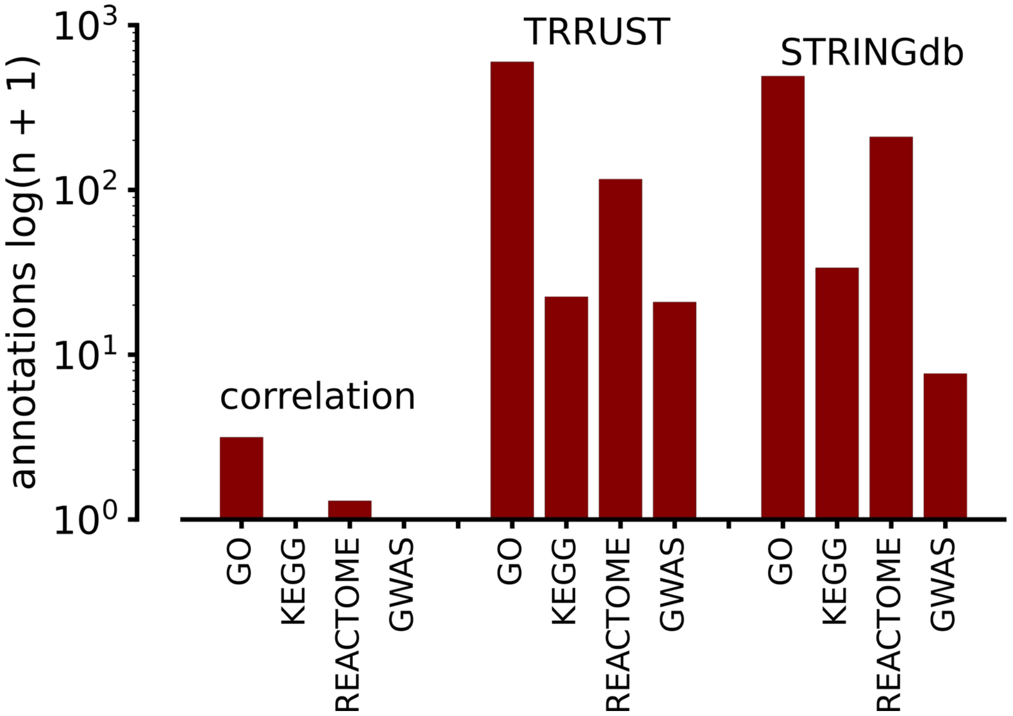

Fig. S3: The number of false positive interactions of different target mapping and annotation methods. We noted that the target mappings based on correlation gave considerably fewer false positive identifications.

Moreover, the GO terms identified in the correlation-based mappings were predominantly related to gene transcription, with just two GO terms accounting for 57% of all the identified terms ('mRNA splicing, via spliceosome', and 'mRNA processing'). We note that the input genes were all TFs, and speculate that these identifications predominantly stem from genes that are involved to the transcriptional process. From this analysis, we decided to only include the correlation-based approach with TFTenricher, but implemented the TF-to-target mapping as a module that easily can be defined by the user.

References

Han et al.: TRRUST v2: an expanded reference database of human and mouse transcriptional regulatory interactions. Nucleic Acids Res. 2018 Jan 4;46(D1):D380-D386. Doi: 10.1093/nar/gkx1013. PMID: 29087512; PMCID: PMC5753191.

Lachmann et al.: Massive mining of publicly available RNA-seq data from human and mouse. Nature Communications 9. Article number: 1366 (2018), doi:10.1038/s41467-018-03751-6

Marbach et al.: Wisdom of crowds for robust gene network inference. Nature methods vol. 9, 8 796-804. (2012), doi:10.1038/nmeth.2016

Szklarczyk et al.: STRING v11: protein-protein association networks with increased coverage, supporting functional discovery in genome-wide experimental datasets. Nucleic Acids Res. 2019 Jan; 47:D607-613
